# Mitral annular plane systolic excursion and intra-myocardial haemorrhage in acute myocardial infarction

**DOI:** 10.1186/1532-429X-17-S1-P163

**Published:** 2015-02-03

**Authors:** Pankaj Garg, Ananth Kidambi, David P Ripley, Laura E Dobson, Peter P Swoboda, Tarique A Musa, Bara Erhayiem, Adam K McDiarmid, John P Greenwood, Sven Plein

**Affiliations:** Multidisciplinary Cardiovascular Research Centre & The Division of Cardiovascular and Diabetes Research, Leeds Institute of Genetics, Health & Therapeutics, University of Leeds, Leeds, UK

## Background

Mitral annular plane systolic excursion (MAPSE) is known to have prognostic importance in the risk stratification of patients with acute myocardial infarction (MI). In post-MI patients with MAPSE<8mm, the combined mortality and hospitalization incidence is 43.8%. Similarly, CMR studies have shown that intra-myocardial haemorrhage (IMH) in the infarct core is an independent marker of prognosis. We hypothesised that the MAPSE on 4-chamber cine-CMR is correlated to left ventricular ejection fraction (EF) and to the presence of IMH.

## Methods

**Figure 1:**
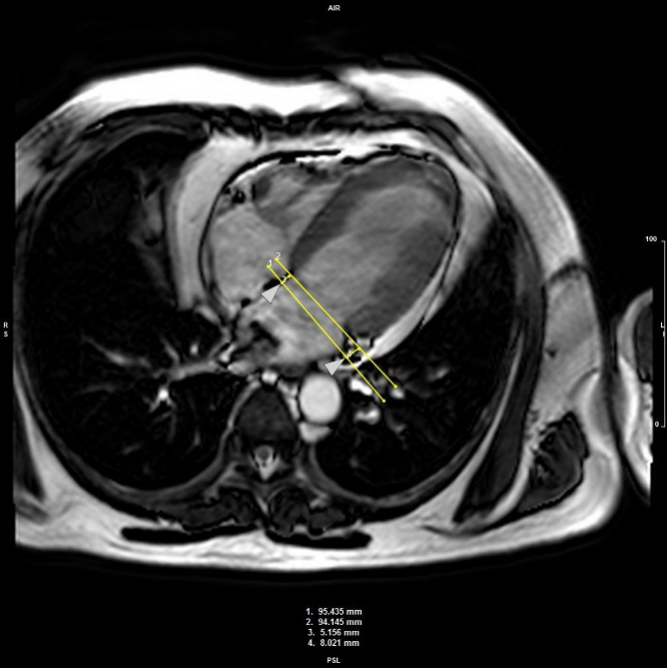
Illustration to show how the MAPSE measurements were taken in the four-chamber cine using contour forwarding.

Fourty-four patients received CMR examination at 3T (Intera CV, Philips Healthcare, Best, The Netherlands) within 3 days following reperfused ST-elevation acute MI. Cine, T2-weighted, T2* imaging and LGE imaging (0.1 mmol/kg gadolinium DTPA) were performed. Infarct and microvascular obstruction (MO) extent were measured from late gadolinium enhancement (LGE) images. The presence and extent of IMH was investigated by combined analysis of T2w and T2* sequences. We computed MAPSE (medial, lateral and average) using the 4-chamber cine. A line was drawn across both the medial and lateral mitral annulus as a reference point in end-diastole (just after closure of mitral valve). A second reference line was drawn across the same plane on an image taken just after closure of the aortic valve. The longitudinal distance parallel to the left ventricular wall was measured for both medial and lateral walls (Figure 1).

## Results

**Figure 2:**
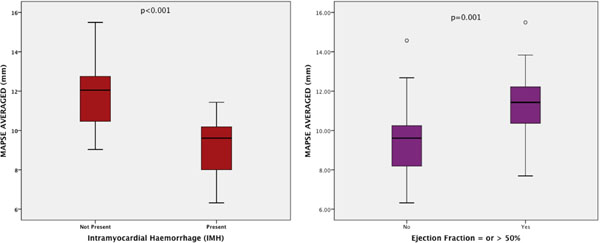
Box-plot of averaged-MAPSE to IMH and EF.

Of the 44 patients, 37 (84%) were male. Mean age of our studied population was 58.27±11.41. CMR parameters were as follows: LVEF 48.2±11.4%; indexed left ventricular end-diastolic volume (LVEDVi) 82.4±15.7 ml/m2; infarct volume LGE of 15.5±12.2 ml; medial MAPSE of 9.8±2.9 mm; lateral MAPSE of 10.97±2.3mm and averaged MAPSE of 10.27±2.1mm. Lateral MAPSE was significantly reduced to previously studied age-adjusted normal values (12.8±2.2mm; p=0.0087)(1).Controlling for age/gender/hypertension /hypercholesterolemia/smoker/diabetes, IMH was strongly negatively correlated to average MAPSE (r=-0.65; p<0.001). Averaged MAPSE was also moderately correlated to LVEF (r=0.47; p=0.001) (Figure 2). A cut-off value of 11mm for averaged MAPSE is 96% sensitive and 68.4% specific in ruling out IMH.

## Conclusions

Averaged MAPSE, which is a simple CMR derived parameter of longitudinal function, has the potential to predict the presence of IMH in the setting of re-perfused acute myocardial infarction. This parameter could be easily measured at bedside by transthoracic echocardiography to predict presence of IMH.

## Funding

JPG and SP receive a research grant from Philips Healthcare. SP is funded by British Heart Foundation fellowship (FS/10/62/28409).
